# Supplementary material for: Six-Year Prescription Pattern of Antimicrobial Use in Cats at the Veterinary Teaching Hospital of the University of Pisa
Source: Animals (Basel). 2024 Feb 5;14(3):521. doi: 10.3390/ani14030521 (PMC10854698; doi:10.3390/ani14030521)
Supplement: Supplementary file 1 [file animals-14-00521-s001.zip › animals-2794309-supplementary.pdf]

Table S1. Complete information of distribution of antimicrobial classes prescribed from 2017 through 2022. Percentages were calculated on the total prescribed antimicrobial classes per year. * $p < 0.05$; ** $p < 0.01$; *** $p < 0.001$; **** $p < 0.0001$.

	**2017 n (%)**	**2018 n (%)**	**2019 n (%)**	**2020 n (%)**	**2021 n (%)**	**2022 n (%)**	**Tot 6 y n (%)**	***p*-Value Trend**
**Aminoglycosides**	**6 (3.6)**	**11 (4.3)**	**5 (5.4)**	**1 (3.4)**	**2 (3.7)**	**1 (1.3)**	**26 (3.9)**	**<0.001**
Tobramycin	3 (1.8)	9 (3.5)	3 (3.3)	1 (3.4)	2 (3.7)	1 (1.3)	19 (2.8)	
Gentamicin	2 (1.2)	1 (0.4)	1 (1.1)	-	-	-	4 (0.6)	
Paromomycin	-	-	1 (1.1)	-	-	-	1 (0.1)	
Neomycin	1 (0.6)	1 (0.4)	-	-	-	-	2 (0.3)	
**Cephalosporins**	**20 (11.9)**	**25 (9.8)**	**9 (9.7)**	**1 (3.4)**	**0 (0.0)**	**2 (2.6)**	**57 (8.4)**	**<0.0001**
Cephalexin	12 (7.2)	11 (4.3)	5 (5.4)	1 (3.4)	-	1 (1.3)	30 (4.4)	
Cefadroxil	4 (2.4)	4 (1.6)	-	-	-	0 (0.0)	8 (1.2)	
Ceftriaxone	4 (2.4)	10 (3.9)	4 (4.3)	-	-	-	18 (2.7)	
Cefovecin	-	-	-	-	-	1 (1.3)	1 (0.1)	
**Fixed-dose combination**	**79 (47.3)**	**103 (40.2)**	**50 (54.3)** *	**13 (44.8)**	**27 (50.0)**	**51 (66.2)** ****	**323 (47.9)**	**<0.0001**
Amoxicillin-clavulanic acid [a]	49 (29.3)	76 (29.7)	48 (52.2)	13 (44.8)	27 (50.0)	51 (66.2) ****	264 (39.1)	
Bacitracin-neomycin sulfate [b]	1 (0.6)	-	-	-	-	-	1 (0.1)	
Chloramphenicol-Colistimethate Sodium-Rolitetracycline [c]	-	5 (2.0)	-	-	-	-	5 (0.7)	
Spiramycin-metronidazole [d]	29 (17.4)	22 (8.6)	2 (2.2)	-	-	-	53 (7.9)	
**Fluoroquinolones**	**51 (30.5)**	**83 (32.4)** **	**21 (22.8)** ****	**11 (37.9)**	**15 (27.8)**	**20 (26.0)**	**201 (29.8)**	**<0.0001**
Enrofloxacin	45 (26.9)	71 (27.7)	16 (17.4)	10 (34.5)	15 (27.8)	12 (15.6)	169 (25.0)	
Marbofloxacin	6 (3.6)	6 (2.3)	4 (4.3)	1 (3.4)	-	7 (9.1)	24 (3.6)	
Orbifloxacin	-	-	-	-	-	1 (1.3)	1 (0.1)	
Ofloxacin	-	4 (1.6)	-	-	-	-	4 (0.6)	
Pradofloxacin	-	2 (0.8)	1 (1.1)	-	-	-	3 (0.4)	
**Fusidic Acid**	**1 (0.6)**	**2 (0.8)**	**0 (0.0)**	**0 (0.0)**	**0 (0.0)**	**0 (0.0)**	**3 (0.4)**	**<0.05**
**Lincosamides**	**2 (1.2)**	**6 (2.3)**	**1 (1.1)**	**0 (0.0)**	**1 (1.9)**	**1 (1.3)**	**11 (1.6)**	**<0.05**
Clindamycin	2 (1.2)	6 (2.3)	1 (1.1)	-	1 (1.9)	1 (1.3)	11 (1.6)	
**Nitroimidazoles**	**0 (0.0)**	**9 (3.5)** ****	**0 (0.0)**	**0 (0.0)**	**0 (0.0)**	**0 (0.0)**	**9 (1.3)**	**<0.01**
Metronidazole	-	9 (3.5)	-	-	-	-	9 (1.3)	
**Penicillins**	**1 (0.6)**	**0 (0.0)**	**0 (0.0)**	**0 (0.0)**	**0 (0.0)**	**0 (0.0)**	**1 (0.1)**	0.11
Ampicillin	1 (0.6)	-	-	-	-	-	1 (0.1)	
**Phenicols**	**1 (0.6)**	**1 (0.4)**	**0 (0.0)**	**0 (0.0)**	**0 (0.0)**	**0 (0.0)**	**2 (0.3)**	0.07
Chloramphenicol	1 (0.6)	1 (0.4)	-	-	-	-	2 (0.3)	
**Polymyxins**	**1 (0.6)**	**3 (1.2)**	**2 (2.2)**	**1 (3.4)**	**0 (0.0)**	**0 (0.0)**	**7 (1.0)**	0.07
Polymyxin B	1 (0.6)	3 (1.2)	2 (2.2)	1 (3.4)	-	-	7 (1.0)	

**Tetracyclines**	**5 (3.0)**	**13 (5.1)**	**4 (4.3)**	**2 (6.9)**	**9 (16.7)** *	**2 (2.6)** *	**35 (5.2)**	0.11
Doxycycline	5 (3.0)	13 (5.1)	3 (3.3)	2 (6.9)	9 (16.7)	2 (2.6)	34 (5.0)	
Tetracycline	-	-	1 (1.1)	-	-	-	1 (0.1)	
**Total**	**167 (100)**	**256 (100)** ****	**92 (100)** ****	**29 (100)** ****	**54 (100)** **	**77 (100)** *	**675 (100)**	**<0.0001**

[a] Synulox; [b] Bimixin; [c] Colbiocin; [d] Stomorgyl

Table S2. Complete information of antimicrobial prescriptions by route in the total 6-year period. Percentages were calculated on the total 6-year prescriptions.

	Route of administration		
	Oral	Parenteral	Topical
	n (%)	n (%)	n (%)
**Aminoglycosides**	**1 (0.1)**	**0 (0.0)**	**25 (3.7)**
Tobramycin	-	-	19 (2.8)
Gentamicin	-	-	4 (0.6)
Paromomycin	1 (0.1)	-	
Neomycin	-	-	2 (0.3)
**Cephalosporins**	**38 (5.6)**	**19 (2.8)**	**0 (0.0)**
Cephalexin	30 (4.4)	-	-
Cefadroxil	8 (1.2)	-	-
Ceftriaxone	-	18 (2.7)	-
Cefovecin	-	1 (0.1)	-
**Fixed-dose combinations**	**306 (45.3)** ****	**12 (1.8)**	**5 (0.7)**
Amoxicillin-clavulanic acid [a]	252 (37.2)	12 (1.8)	-
Bacitracin- neomycin sulfate [b]	1 (0.1)	-	-
Chloramphenicol-Colistimethate Sodium-Rolitetracycline [c]	-	-	5 (0.7)
Spiramycin-metronidazole [d]	53 (7.9)	-	-
**Fluoroquinolones**	**186 (27.4)** ****	**10 (1.5)**	**5 (0.7)**
Enrofloxacin	164 (24.1)	5 (0.7)	-
Marbofloxacin	19 (2.8)	5 (0.7)	-
Orbifloxacin	-	-	1 (0.1)
Ofloxacin	-	-	4 (0.6)
Pradofloxacin	3 (0.4)	-	-
**Lincosamides**	**11 (1.6)**	**0 (0.0)**	**0 (0.0)**
Clindamycin	11 (1.6)	-	-
**Penicillins**	**1 (0.1)**	**0 (0.0)**	**0 (0.0)**
Ampicillin	1 (0.1)	-	-
**Phenicols**	**0 (0.0)**	**0 (0.0)**	**2 (0.3)**
Chloramphenicol	-	-	2 (0.3)
**Polymyxins**	**0 (0.0)**	**0 (0.0)**	**7 (1.0)**
Polymyxin B	-	-	7 (1.0)
**Nitroimidazoles**	**9 (1.3)**	**0 (0.0)**	**0 (0.0)**
Metronidazole	9 (1.3)	-	-

**Tetracyclines**	**36 (5.3)**	**0 (0.0)**	**1 (0.1)**
Doxycycline	34 (5.0)	-	-
Tetracycline	-	-	1 (0.1)
**Others**	**0 (0.0)**	**0 (0.0)**	**3 (0.4)**
Fusidic acid	-	-	3 (0.4)
**Total**	**586 (86.8)** ****	**41 (6.1)**	**48 (7.1)**

[a] Synulox; [b] Bimixin; [c] Colbiocin; [d] Stomorgyl
